# Environmental Isolation of *Sporothrix brasiliensis* in an Area With Recurrent Feline Sporotrichosis Cases

**DOI:** 10.3389/fcimb.2022.894297

**Published:** 2022-05-12

**Authors:** Vanessa Brito Souza Rabello, Fernando Almeida-Silva, Bruno de Souza Scramignon-Costa, Beatriz da Silva Motta, Priscila Marques de Macedo, Marcus de Melo Teixeira, Rodrigo Almeida-Paes, Laszlo Irinyi, Wieland Meyer, Rosely Maria Zancopé-Oliveira

**Affiliations:** ^1^ Laboratório de Micologia, Instituto Nacional de Infectologia Evandro Chagas, Fundação Oswaldo Cruz, Rio de Janeiro, Brazil; ^2^ Laboratório de Pesquisa Clínica em Dermatologia Infecciosa, Instituto Nacional de Infectologia Evandro Chagas, Fundação Oswaldo Cruz, Rio de Janeiro, Brazil; ^3^ Núcleo de Medicina Tropical, Faculdade de Medicina, Universidade de Brasília (UnB), Brasília, Brazil; ^4^ Molecular Mycology Research Laboratory, Centre for Infectious Diseases and Microbiology, Faculty of Medicine and Health, Sydney Medical School, Westmead Clinical School, Marie Bashir Institute for Infectious Diseases and Biosecurity, University of Sydney, Westmead Hospital-Research and Education Network, Westmead Institute for Medical Research, Sydney, NSW, Australia; ^5^ Curtin Medical School, Curtin University, Perth, WA, Australia

**Keywords:** *Sporothrix brasiliensis*, environment, sporotrichosis, timber wood, phylogenetic analysis, Brazil

## Abstract

Sporotrichosis has been expanding throughout the Brazilian territory in recent years. New outbreaks have emerged, and consequently, the sporotrichosis agents, mainly *Sporothrix brasiliensis*, should remain in the environment somehow. Therefore, the aim of this study was to investigate the presence of *Sporothrix* spp. in the environment from an area of ​​the Rio de Janeiro state, Brazil, with recurrent cases of human and animal sporotrichosis. Abandoned demolition timber wood samples were collected in the garden of a house where the cases of human and feline sporotrichosis have occurred in the last 10 years. The environmental survey revealed a *Sporothrix* spp. colony from the serial dilution cultures of one abandoned demolition wood sample. In addition, a fungal strain isolated from a cat with skin lesions that lived in the house was also included in the study. The species-specific PCR, and calmodulin partial sequencing identified the environmental and cat isolates as *S. brasiliensis*. Furthermore, the phylogenetic analysis performed with the partial sequences of internal transcribed spacer region and constitutive genes (calmodulin, β-tubulin, and chitin synthase) showed high similarity between environmental and cat isolates from the same geographic region. Moreover, the antifungal susceptibility test revealed that the minimal inhibitory concentration of itraconazole from the environment isolate was lower than the cat isolate, while amphotericin B and terbinafine were similar. Our results show that *S. brasiliensis* is able to maintain itself in the environmental material for years. With this, we corroborate that the eco-epidemiology of sporotrichosis is not well understood, and despite the major occurrence of *S*. *brasiliensis* in Brazil, it is rarely isolated from the environment.

## Introduction

Sporotrichosis is a subcutaneous mycosis caused by the thermo-dimorphic fungi of the genus *Sporothrix*. The genus contains at least 51 species that live as saprobes on soil, decaying wood, and plant debris in environments with high humidity (~90%) (Ramírez-Soto et al., 2018). Most *Sporothrix* species are non-pathogenic, but seven species may cause human infection; those include *Sporothrix schenckii*, *S*. *brasiliensis*, *S*. *globosa*, *S*. *luriei*, *S. mexicana, S. pallida, and S. chilensis. These species are able to* infect warm-blooded hosts *since* they are able to develop the process called dimorphism, changing from their saprophytic filamentous stage at ambient temperature to the parasitic yeast form at 35°C–37°C. Among the pathogenic species, *S*. *schenckii*, *S*. *brasiliensis*, *S*. *globosa*, and *S*. *luriei* are often isolated from humans and animals; the remaining species rarely cause disease, being commonly isolated from environmental sources (Marimon et al., 2007; Rodrigues et al., 2016; Rodrigues et al., 2020; Valeriano et al., 2020). Sporotrichosis occurs after traumatic inoculation *with organic matter harboring these fungi. The main clinical manifestation in humans is the lymphocutaneous form, followed by the fixed cutaneous form, restricted to the local trauma area. Systemic infections are mainly developed in immunocompromised patients (* Zancope-Oliveira et al., 2011 *;* Orofino-Costa et al., 2017 *).*

The largest outbreak associated to an environmental source occurred at a gold mine in South Africa, between 1938 and 1949, with the description of more than 3,000 cases. The woods used to support the mines were identified as the source of the infection (Helm and Bermam, 1947; Quintal, 2000). Another sporotrichosis outbreak involved 84 sapronotic transmission cases reported in 1988, affecting the people who were exposed to *Sphagnum* moss used to protect and moisten the roots of the tree seedlings in 15 states of the United States (Dixon et al., 1991). In 2011, another outbreak in a gold mine in South Africa was reported, where patients were infected by *S*. *schenckii*, while environmental isolates were identified as *S*. *mexicana*. Despite the difference between the species, the probable source of infection was contaminated soil and untreated woods at the gold mine (Govender et al., 2015).

Although the environmental outbreaks related to human sporotrichosis were well described in different geographic regions, there is little knowledge about the ecology of the clinically relevant species related to them. *S. schenckii* is often found from environmental sources from different countries, including Brazil, Argentina, the United States, Germany, Italy, China, and India (Ramírez-Soto et al., 2018). However, other clinical species are rarely isolated from the environment, such as *S. luriei* and *S*. *brasiliensis*. The latter is the main species described in Brazil, causing important zoonotic outbreaks in different regions of the country (Gremião et al., 2020; Rodrigues et al., 2020). Therefore, the knowledge of *Sporothrix* spp. ecology will provide useful information to support public health management. To address this issue, the current study carried out an environmental survey in an area within a city of Rio de Janeiro state, Brazil, where recurrent cases of the sporotrichosis were reported.

## Materials and Methods

In 2020, the samples of abandoned demolition woods, from a tree from the Class Magnoliopsida, which include some species of the genera *Paubrasilia*, *Jacaranda*, *Manilkara*, and others that are extensively used in Brazilian building constructions, were collected from a house in Petrópolis, Rio de Janeiro State, Brazil (22°31’51.3”S 43°10’38.2”W), which had a pet cat with sporotrichosis confirmed by the fungal culture in 2017, and other probable human and feline sporotrichosis cases, which occurred in the last 10 years. A pool of environmental samples was collected by friction on the wood surfaces with a sterile transport swab with Amies medium, added with charcoal (ABSORVE, São Paulo, Brazil), and transported at room temperature to the Mycology Laboratory of the Evandro Chagas National Institute of Infectious Diseases (INI/FIOCRUZ). Two swab samples from abandoned woods deposited in distinct locations of the house and wood fragments were collected, with a sterile scalpel, and stored in 15 ml Falcon tubes.

Serial dilutions in sterile distilled water were performed with the swab samples. First, the swabs were vigorously mixed in 300 µl sterile distilled water and ten-fold dilutions were made up to the 10^-5^. Each dilution was cultured in duplicate on potato dextrose agar (PDA; Becton Dickinson and Company Sparks, MD, USA) and incubated at 25°C for at least 30 days. Colonies that presented macro- and micromorphology suggestive of *Sporothrix* spp. were subcultured on PDA and thermo-dimorphism was evaluated on brain heart infusion agar (BHI; Becton Dickinson and Company Sparks, MD, USA) at 37°C for 7 days.

The fungal strain isolated from a cat with skin lesions compatible with sporotrichosis that lives in the house where the wood samples was collected was also included in the study. The owner allowed to include the cat in the study, so the fragments of the skin lesions were obtained by biopsy and cultured on Sabouraud dextrose, Mycosel, and BHI agars (Becton Dickinson GmbH). After isolation, the fungal strain was stored at -80°C.

DNA extraction directly from wood fragments was conducted as described by Macedo et al. (2020), using the DNeasy^®^ PowerSoil^®^ Kit (Qiagen, Hilden, Germany). DNA extraction from *Sporothrix* isolates was performed from the filamentous form of the fungus according to Muniz et al. (2010), with the following modifications: the lysis buffer contained 1M Tris pH 8, 50 mM EDTA, and 20% sucrose, and the DNA was precipitated in 100% ethanol with 3 M sodium acetate.

The DNA extracted directly from the wood samples, from putative *Sporothrix* spp. colonies and from the cat isolate, were used as templates in a species-specific PCR according to Rodrigues et al. (2015). The primers for the three major pathogenic *Sporothrix* species (*S. brasiliensis*, *S. globosa*, and *S. schenckii*) were used in the reactions. The environmental DNA was tested in triplicate, to improve fungal detection odds.

A partial sequencing of the constitutive genes calmodulin, chitin synthase, and β-tubulin (Marimon et al., 2007), and the internal transcribed spacer region (ITS1/2) (Zhou et al., 2014) from clinical and environmental *Sporothrix* isolates were performed in order to conduct a phylogenetic analysis. Automated sequencing was done using the FIOCRUZ Technological Platforms. The sequences from both DNA strands were edited with the software Sequencher version 4.9 and aligned by MEGA version 7. Additional sequences of *Sporothrix* spp. deposited in the GenBank (**Table 1**) were included in the phylogenetic analysis generating a maximum likelihood (ML) tree with 1,000 bootstrap replications to estimate the branch confidence values showed on each branch.

**Table 1:** Isolates and GenBank Identification.

Isolate	Species	CAL	CHS	Bt2	ITS
**CBS 120339 (IPEC 16490)**	*S. brasiliensis*	AM116899	AM117417	AM116946	KP017087
CFP 01022 (IPEC 19536)^*^	*S. brasiliensis*	ON014839	ON014840	ON014841	OM949881
CFP 01043^*^	*S. brasiliensis*	MZ670750	MZ670752	MZ670754	MZ576443
CFP 01042^*^	*S. brasiliensis*	MZ670751	MZ670753	MZ670755	MZ576444
**CBS 359.36**	*S. schenckii*	AM117437	AM114872	AM116911	KP017100
**FMR 8597**	*S. globosa*	AM116907	AM117426	AM116964	FN549904
**CBS 937.72**	*S. luriei*	AM747302	AM748698	AM747289	AB128012
**CBS 302.73**	*S. pallida*	AM398396	AM748692	AM498343	KP017078
**CBS 120341**	*S. mexicana*	AM398393	AM748696	AM498344	FN549906

*Sequences generated in this study, CFP 01043 wood isolate and CFP 01043 cat isolate.

CFP, Collection of Pathogenic Fungi, Instituto Nacional de Infectologia Evandro Chagas, Fundação Oswaldo Cruz, Rio de Janeiro, Brazil; IPEC, Instituto de Pesquisa Clínica Evandro Chagas, FIOCRUZ, Rio de Janeiro, Brazil; CBS, Centraalbureau voor Schimmelcultures, Utrecht, The Netherlands; FMR, Facultat de Medicina i Cien`cies de la Salut, Reus, Spain; CAL, calmodulin gene; CHS, chitin synthase gene; Bt2, β-tubulin gene; ITS, internal transcribed spacer region.

The antifungal agents assessed were itraconazole (ITR), terbinafine (TRB), and amphotericin B (AMB). The serial dilutions of antifungal agents were prepared in dimethyl sulfoxide, and the different working concentrations of antifungal drugs, ranging from 0.015 to 8 mg/L, were distributed in 96-well microplates. The conidial suspension (1–5 × 10^4^/ml) from 7-day-old *Sporothrix* spp. cultures on PDA at 35°C was prepared in 3 ml of 0.9% sterile saline solution. The susceptibility test was performed using a broth microdilution assay according to the M38-A2 CLSI reference guidelines (CLSI - Clinical and Laboratory Standards Institute, 2008). The *Sporothrix* suspension was diluted 1:50 in the RPMI 1640 medium buffered with 0.165 mol/L morpholinepropanesulfonic acid (pH 7.0), and 100 µl of each isolate were added to the wells of 96-well microplates. The fungal inoculum without any antifungal agent was used as growth controls, and the sterility controls consisted of only the RPMI medium without fungus or antifungal drugs. The reference strains *Aspergillus fumigatus* ATCC 204305 and *Aspergillus flavus* ATCC 204304 were used as the quality controls of each assay. All tests were performed at least in duplicate; MICs were determined by visual inspection after 48–72 h of incubation at 35°C, as described (CLSI - Clinical and Laboratory Standards Institute, 2008). The MIC of itraconazole and amphotericin B was determined as the lowest concentrations that completely inhibited fungal growth, and for TRB, it was the lowest concentration that resulted in at least 80% reduction in growth.

## Results

**Figure 1:**
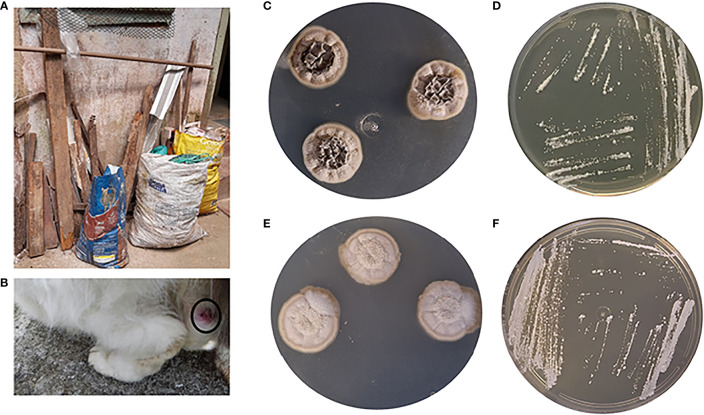
**(A)** Wood disposal in the garden of the studied house. **(B)** Cat with sporotrichosis. **(C, D)** *S*. *brasiliensis* isolated from wood. **(E, F)** *S*. *brasiliensis* isolated from cat. **(C, E)** Filamentous form (13 days at 25°C). **(D, F)** Yeast form (7 days at 37°C).

The skin lesion fragments from the cat (**Figure 1B**) yielded colonies with macro- and micromorphology compatible with *Sporothrix* spp. This cat was treated with itraconazole (100 mg/day) for 3 months, with apparent healing of the lesion. However, after this period, his injuries and general condition progressively worsened. The cat was admitted to a veterinary clinic and died some weeks later. At this point, the fungal isolate from this animal was stored at -80°C.

Three years later, an environmental survey was conducted in the cat´s house (**Figure 1A**) and just one putative *Sporothrix* spp. colony from the 10^-4^ serial dilution of one abandoned demolition wood grew on PDA at 25°C around 28 days after inoculation (**Figure 1C**); in the others, serial dilution was observed in the growth of saprobic fungi or other microorganisms. The *Sporothrix* spp. strain from the cat (**Figure 1E**) was re-grown and evaluated together with the environmental isolate. Both presented conversion to the yeast phase on BHI agar at 37°C, which confirmed their dimorphism (**Figures 1D, F**).

**Figure 2:**
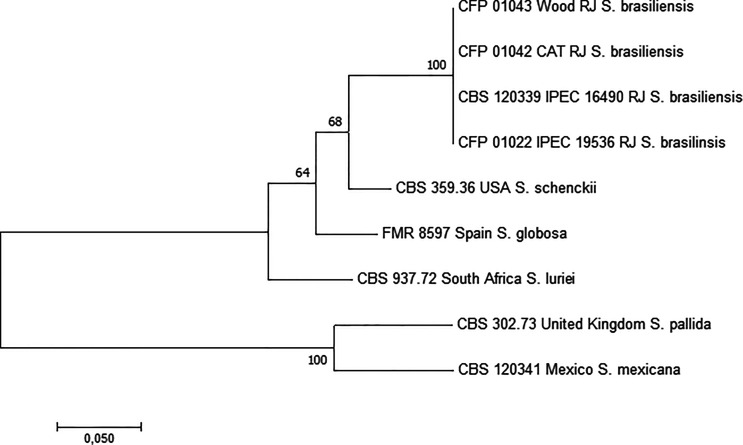
Phylogenetic tree of *Sporothrix* spp., including the studied *S. brasiliensis* isolates studied herein, obtained by ML analysis based on partial sequences of the calmodulin, chitin synthase, β-tubulin genes, and internal transcribed spacer (ITS1, 5.8s, ITS2). The 1,000 bootstrap values are represented on the branches. RJ, Rio de Janeiro state, Brazil.

*Sporothrix* DNA was not detected using species-specific PCR primers for *S. brasiliensis*, *S. schenckii*, or *S. globosa* in the total DNA extracted direct from the wood scrapings. The environmental and cat isolates were identified as *S*. *brasiliensis* by species-specific PCR and partial calmodulin sequencing. Moreover, the phylogenetic analysis performed with partial calmodulin, chitin synthase, the β-tubulin gene, and ITS region sequence showed 100% similarity between the environmental and the cat isolates from the same region (**Figure 2**).

The MIC values from the environmental and cat isolates are described in **Table 2**. In summary, the MIC of TRB was lower than those observed for the other drugs from both isolates. Moreover, the susceptibilities in strains from these two sources were similar for TRB and AMB. The MIC of ITR from the environmental isolate was at two-fold dilutions lower than that observed for the cat isolate.

**Table 2:** MICs (mg/L) of the *S. brasiliensis* strains evaluated in this study.

Isolates	ITR	TER	AMB
Wood	0.25	0.06	1.0
Cat	1.0	0.06	2.0

ITR, itraconazole; TRB, terbinafine; AMB, amphotericin B.

## Discussion

During the major sporotrichosis outbreak in South Africa, *S. schenckii* was found in the timber supporting the mines, confirming it as the common environmental source of the infections of more than 3,000 miners (Quintal, 2000). The present work shows that *S. brasiliensis* may also be encountered in timber samples, which can act as environmental sources of infection for humans and animals.

The eco-epidemiology of the sporotrichosis agents is not well known. Moreover, despite the major occurrence of *S*. *brasiliensis* in Brazil, it is rarely isolated from the environment. Some authors attribute this factor to the low concentration of clinically relevant *Sporothrix* species in the environment (Ramírez-Soto et al., 2018; Rodrigues et al., 2020). However, the high sporotrichosis incidence in Brazil raises the possibility that the environmental *Sporothrix* burden is higher as hypothesized. A study revealed, by whole genome sequencing, that strains from Southeast Brazil, where *S. brasiliensis* first emerged, and Mid-Western Brazil, where *S. brasiliensis* emerged more recently, appear to have separated around two million years ago, supporting different environmental niches for these genotypes (Eudes Filho et al., 2020).

If the environmental *Sporothrix* burden is very high, the uncommon isolation of *Sporothrix* spp. from nature may be related to the limitations of the available detection methods or the paucity of environmental studies on the sporotrichosis agents. Poester et al. (2018) evaluated the presence of *Sporothrix* spp. in soil samples from places in Southern Brazil where zoonotic sporotrichosis also occurs. Despite the 101 samples collected from the residence of cats with sporotrichosis, none of the cultures were positive for *Sporothrix* spp., which could be related to the moderate growth of *Sporothrix* spp, making their detection in soil samples difficult due to the abundant growth of numerous other saprobic microorganisms (Poester et al., 2018). Rodrigues et al. (2014), who did not recover any culture morphologically resampling *Sporothrix* spp. after a direct plating of soil samples, also observed this. In fact, several filamentous fungi, such as *Acremonium*, *Aspergillus*, *Fusarium*, and *Penicillium*, grow faster than *Sporothrix* in culture, so they can dominate the culture media before *Sporothrix* species appear (Ramírez-Soto et al., 2018). Thus, the success of the *Sporothrix* isolation herein reported may be associated to the method of processing the swab culture with an important step of serial dilutions of the sample, reducing the number of other fungi. Moreover, the growth of the environmental sample on PDA could stimulate fungal sporulation, facilitating its isolation. On the other hand, the PDA medium may not inhibit the undesirable fungi, but it also does not hinder the growth of *Sporothrix* spp.

Human and animal cases of sporotrichosis occurred over the last 10 years in the studied house. It is interesting to note that these cases started to occur after a garden area was transformed in a rubbish disposal site. It has been described that sporotrichosis is associated in Brazil with areas of poor sanitary conditions (Silva et al., 2012; Alzuguir et al., 2020). Two hypotheses may explain the occurrence of *S. brasiliensis* in the studied house: the demolition woods already had *S. brasiliensis* that encountered mammal hosts after disposal or the woods became contaminated with the fungus after a case of sporotrichosis in a cat, which present a high fungal burden, that could have been in contact with woods. Either way, our results show that *S. brasiliensis* is able to maintain itself in this environmental material for years. This is reinforced by the isolation of genetically related strains 3 years apart and by the reports of neighbours that some cats with access to this area presented with skin lesions suggestive of sporotrichosis.

Our results also suggest that *S*. *brasiliensis* infections may occur by sapronotic transmission, and it does not occur exclusively *via* zoonotic transmission as has been previously reported (Rodrigues et al., 2020; Rossow et al., 2020). This is supported by the fact that the *S*. *brasiliensis* genotype from the environment was identical to that of the cat isolate. Our data are similar to the studies described in Argentina, where many cats with sporotrichosis by *S*. *brasiliensis* were rescued from abandoned old houses, which contained dirty pine wood floors as a probable source of infection (Etchecopaz et al., 2021). Furthermore, a woodworker also from Argentina acquired the fungus *via* the traumatic inoculation of a pine wood splinter with *S*. *brasiliensis* being identified from his lesion. However, in these cases, *S*. *brasiliensis* was not isolated from the pine wood (Etchecopaz et al., 2021), in contrast to that found in our study. Moreover, *S. brasiliensis* was identified in Brazil from cat’s feces collected in a sand heap (Montenegro et al., 2014) and from armadillo cave soil samples in Argentina (Etchecopaz et al., 2021).

The isolation of *S*. *schenckii* from the environment was successfully achieved after mice inoculation with a soil sample (Rodrigues et al., 2014). Nevertheless, this method has major issues for routine use since it requires specific laboratory structure for animal housing, it is costly and time consuming, and, more importantly, it has major ethical aspects related to the use of experimental vertebrate animals. Therefore, other methods need to be used. Molecular methods are emerging as powerful tools for the detection of pathogenic fungi in environmental samples. Species-specific PCR was developed for the identification of *Sporothrix* species in pure cultures or in infected tissues, as observed in an experimental murine model of infection (Rodrigues et al., 2015). In the present study, this method was inefficient to detect *S. brasiliensis* DNA in the environmental material. Poester et al. (2018) also observed negative DNA by species-specific PCR and nested PCR for the five *Sporothrix* species (*S*. *brasiliensis*, *S*. *schenckii*, *S*. *globosa*, *S*. *mexican*a, and *S*. *pallida*) from soil samples. It is expected that hundreds of microorganisms co-exist with the pathogenic *Sporothrix* species in the environment, so probably the use of more sensitive methods, capable to detect small amounts of *Sporothrix* DNA among high amounts of DNA from other microorganisms, is necessary for environmental molecular studies for the agents of sporotrichosis. Another problem that would explain the undetected DNA is that PCR inhibitors are present in environment samples, requiring a massive improvement of this method.

The cat was treated with itraconazole, and the *in vitro* susceptibility of the isolate showed good result for this drug. Therefore, the death of the cat may be related to its immune system or other unknown diseases. Furthermore, the earlier the treatment is started, the higher the chance of cure. In addition, the sporotrichosis treatment is too long mainly for cats, so it is very important to follow it strictly (Gremião et al., 2017). The indiscriminate use of antifungals may lead to the emergence of resistance mechanism drugs. Antifungal resistance is a worldwide challenge mainly for treating invasive fungal infections (Wiederhold, 2017; Fisher et al., 2022). The environmental MIC for ITR was lower than the clinical isolate; however, the number of isolates available in this study is small to make comparisons, but this should be appraised in future studies not only with *Sporothrix* isolates but with other pathogenic fungi as well.

Furthermore, it is important to highlight the issues related to the inadequate disposal of waste observed in the house studied. The demolition wood exposed to rain, humidity, and heat becomes a source for *S. brasiliensis* maintenance in the environment as a potential contamination source for humans and animals. Therefore, education activities are necessary in endemic areas to explain to people how to properly dispose demolition material and to avoid the accumulation of waste and organic matter in inappropriate places. Moreover, it is necessary to know how to treat timber wood to avoid contamination. In the 2011 outbreak of a gold mine in South Africa, it was recommended that all new timber should be treated with tar, making the use of personal protective equipment obligatory, after which, no new cases occurred anymore (Govender et al., 2015). However, some substances to prevent microorganism growth may be toxic for human, animals, and the environment. Therefore, the solution to eliminate the source in the gardens described in this study is not simple but should be discussed since this scenario is common in the hyperendemic area of zoonotic sporotrichosis in Rio de Janeiro State, Brazil (Silva et al., 2012; Alzuguir et al., 2020).

In conclusion, the main agent of sporotrichosis in Brazil, *S*. *brasiliensis*, may be found living in organic matter as saprobes, thus these materials can be a source of infection by this species for human and animals.

## Data Availability Statement

The original contributions presented in the study are included in the article. Further inquiries can be directed to the corresponding author.

## Ethics Statement

Ethical review and approval was not required for the animal study because the study was performed with the clinical data of an animal attended in a routine dignosis for sporotrichosis. The owner of the animal has given permission to use this data after the death of the animal. Written informed consent was obtained from the owners for the participation of their animals in this study.

## Author Contributions

The first draft of the manuscript was written by VR, RA-P, and PM. VR designed and developed the experiments and FA-S, BS-C and BM did part of wood DNA identification. MT, LI, WM, and RZ-O analyzed the data and corrected the manuscript. All authors read and approved the final version of the manuscript.

## Funding

This work was supported in part by Conselho Nacional de Desenvolvimento Científico e Tecnológico [CNPq 302796/2017-7] and Fundação Carlos Chagas Filho de Amparo à Pesquisa do Estado do Rio de Janeiro [FAPERJ E-26/202.527/2019]. Moreover, this study was partially supported by the Coordenação de Aperfeiçoamento de Pessoal de Nível Superior – CAPES - Finance Code 001.

## Conflict of Interest

The authors declare that the research was conducted in the absence of any commercial or financial relationships that could be construed as a potential conflict of interest.

## Publisher’s Note

All claims expressed in this article are solely those of the authors and do not necessarily represent those of their affiliated organizations, or those of the publisher, the editors and the reviewers. Any product that may be evaluated in this article, or claim that may be made by its manufacturer, is not guaranteed or endorsed by the publisher.
